# Feelings of loneliness and isolation: Social brain and social cognition in the elderly and Alzheimer's disease

**DOI:** 10.3389/fnagi.2022.896218

**Published:** 2022-07-22

**Authors:** Rosalba Morese, Sara Palermo

**Affiliations:** ^1^Faculty of Biomedical Sciences, Università della Svizzera italiana, Lugano, Switzerland; ^2^Faculty of Communication, Culture and Society, Università della Svizzera italiana, Lugano, Switzerland; ^3^Department of Psychology, University of Turin, Turin, Italy; ^4^Neuroradiology Unit, Department of Diagnostic and Technology, Fondazione IRCCS Istituto Neurologico Carlo Besta, Milan, Italy

**Keywords:** elderly, loneliness, social isolation, social brain, social cognition, social neuroscience, mental health, Alzheimer's disease

## From attachment styles to interpersonal neurobiology

Interpersonal neurobiology presents an integrated view of the development of the human mind, investigating how this occurs from the mutual influence between human relationships and brain structure and function: the focus of this approach is to understand how the brain gives rise to mental processes and how it is directly shaped by interpersonal experiences (Siegel, 1999). Through this approach we can understand what processes are useful in facilitating cognitive-behavioral development, emotional and psychological wellbeing, and resilience certainly during early childhood but probably throughout life. Underlying the mentioned processes is indeed a fundamental mechanism of integration that can be examined at different levels, from interpersonal to neurological (Siegel, 2001). Interpersonal neurobiology proposes an interpretation of mental processes whose main characteristics are (1) being both embodied, in the body, and relational; (2) the smooth flow of metabolites and information; and (3) the flow of information in the percipient and between people (Siegel, 2001). Knowing how to control and knowing how to modify this flow of energy and information, the basis of healthy regulation, are skills that are acquired in families with secure attachment (Bowlby, 1969; Siegel, 2001; Bowlby and King, 2004).

The child comes into the world genetically programmed to establish attachment bonds with its caregivers who will become, thus, the child's attachment figures (Bowlby, 1969; Cassidy and Shaver, 1999; Bowlby and King, 2004). The attachment system is considered a motivational system: an innate, adaptive, biologically determined system that drives a child to create certain selective attachments in his or her life. Although the attachment system is programmed at the brain level, the experiences a child has throughout his or her childhood go a long way toward shaping that system (Bowlby, 1969; Cassidy and Shaver, 1999; Bowlby and King, 2004). The succession of relational experiences prompts the activation of brain neurons that respond to sensory events from the outside world or internally generated images from the brain itself (Gazzaniga, 1995; Kandel et al., 2012). The process is used to create a mental image, sensory image or linguistic representation of a concept or object (Siegel, 2001; Kandel et al., 2012). According to Siegel (2001), the neural substrate also allows the formation of an emergent self, a proto-self, determined largely by genetic and constitutional characteristics. This sense of self is embedded in the brain as well as in its interactions with the environment: *internal working model* (Siegel, 2001). On the other hand, the child's mind seems to develop a fundamental process in which the other's mental states (the so-called Theory of Mind, ToM) are also represented within the neuronal functioning of the brain (Stone et al., 1998). The sense of acting, coherence, affectivity and even the continuity of the self (memory) are therefore influenced by interaction with others. In this way, experience shapes the function of neural activity and can potentially shape the evolving structure of the brain throughout the lifespan. Developmental stages and aging change the concept of security from childhood's pursuit of physical proximity to sophisticated forms of relating and representing others. Through the process of creating new meanings from memories, internal working models reorganize themselves according to new relational experiences, thereby assisting in constructing a consistent self-model through adulthood (Bowlby, 1969; Cassidy and Shaver, 1999; Bowlby and King, 2004).

The attachment theory of John Bowlby is applicable to every age group, but researchers have been slow to study attachment in older adults (Bradley and Cafferty, 2001). Study findings suggest, however, that attachment issues could be relevant for the elderly, given that aging can be associated with separation, loss, and vulnerability (Bradley and Cafferty, 2001). As a matter of fact, the personal attachment style is associated with a range of outcomes in later life (such as reactions to the loss of a loved one, general wellbeing and adjustment to chronic illness and caregiver burden). The implications of attachment styles and the questions raised by interpersonal neurobiology regarding social isolation and loneliness on the directions of aging and the acceleration of any neurodegenerative process are discussed here.

## Theoretical framework: Social brain and social cognition

Networks of relationships are important to Siegel (2001) since neural networks appear strongly influenced by relations with others, beyond genetic influences. In his words: “human connections shape neural connections, and each contributes to mind. Relationships and neural linkages together shape the mind. It is more than the sum of its parts; this is the essence of emergence” (Siegel, 2001, p. 3). The horizon of interpersonal neurobiology thus allows for a broad perspective that is reflected in neuroimaging investigations. Thus, social neuroscience hypothesizes brain evolution on the level of intersubjective actions. Due to the social environment in which primates live, specific selective pressures have led to the evolution of neurocognitive mechanisms capable of handling the challenges of social interaction (Adenzato and Enrici, 2005). Humans' social cognition consists of psychological processes that allow us to make inferences about what others are thinking and feeling (Adenzato and Enrici, 2005; Adolphs, 2009). The way social information is processed is divided into automatic and stimuli-driven processes, and those that are deliberate and controlled, but sensitive to context and strategy (Adolphs, 2009). In their proposal, the “social brain hypothesis,” Byrne and Whiten (1988) were among the first to argue that complex social environments serve as a dominant selective pressure for human brain size. By appealing to particular pressures that a species adapted to social interactions would have faced, the social brain hypothesis attempts to explain the extraordinary size and complexity of the human brain (Barrett and Henzi, 2005; Dunbar and Shultz, 2007a,b).

In everyday life, social interaction is one of the most complex mental activities in which humans engage. The high cognitive load is necessary to predict the behavior of people involved in social interaction. In particular, the functions involved in the social brain relate to social cognition, which is important for sociability. The term social cognition refers to the set of abilities that enables an individual to construct mental representations of his or her relationships with others and to use these representations to adapt behaviors to the context (Adolphs, 2001). Social groups are complex in nature, and it is their complexity that has led to the advancement of prefrontal brain functioning and specialization (Adenzato and Enrici, 2005). Not only the prefrontal areas but also other cortical and subcortical structures are involved in the processing of social stimuli. Social information activates complex neural circuits that connect cortical and subcortical regions, including those usually thought to be involved in the emotional processing of stimuli, such as the amygdala, as well as those usually thought to be involved in the cognitive processing of stimuli, such as the temporo-occipital junction and the medial prefrontal cortex (Van Overwalle, 2009). This widespread neural involvement reverberates the fact that social cognition is a high-order function. Indeed, social cognition is broad and varied; it refers to all mental processes useful in social interaction, among which the ToM and mentalizing play a significant role. Premack and Woodruff (1978) define ToM as the ability t to attribute to other individuals' mental states that are different from one's own. Mentalisation is an inherently imaginative activity involving the attribution of intentional mental states based on clues. In mentalisation, it is recognized that a person's actions are autonomous and can be explained by his or her internal state (McLaren and Sharp, 2020).

According to Dodich et al. (2015), we deal with a multidimensional process in which different components are integrated. Among these, attribution of emotions and intentions is a very important component during the representation of mental states. Other authors use this term to refer to thinking or feeling about others' mental states (Saxe et al., 2006a,b; Van Overwalle, 2009). Some neural structures, such as the anterior cingulate cortex (ACC) (Palermo, 2017, 2020b), the medial prefrontal cortex (MPFC), the temporoparietal junction (TPJ), the posterior cingulate cortex (PCC), and the superior temporal sulcus (STS) are known to play a role (Adolphs, 2001; Saxe and Kanwisher, 2003). On the other hand, Sebastian et al. (2012) used functional magnetic resonance imaging (fMRI) on healthy subjects which showed that some brain areas involved with mentalization and perspective-taking, like the temporoparietal junction and the ventromedial prefrontal cortex, are recruited when affective stimuli are present.

In fact, it is only one behavioral domain, that of social cognition (Laird et al., 2011), that is strongly and exclusively associated with a neural network that closely resembles the default mode network (DMN), demonstrating bilateral activation of the inferior parietal/TPJ, posterior precuneus/cingulate, and medial frontal (Smith et al., 2009; Mars et al., 2012). Similar conclusions had previously been reached by Schilbach et al. (2008). When they examined the DMNs' responses to different types of cognitive stimulation some activations were quite similar to those observed in various aspects of social cognition: the left angular gyrus/TPJ in differentiating between self and others (Vogeley and Fink, 2003); the anterior cingulate in monitoring action in self and others (Amodio and Frith, 2006); the precuneus in social interactions (Schilbach et al., 2006). According to the authors, the biological “baseline” of the human brain corresponds to a psychological “baseline,” our predisposition to engage in social cognition by default (Schilbach et al., 2008; Mars et al., 2012). Cognitive processes geared toward self-reflection, such as introspection and autobiographical memory, have been also linked to the DMN, and its integrity is now considered crucial to mental health (Grieder et al., 2018).

## Loneliness and its impact on psychological wellbeing and social cognition

Loneliness is a negative emotional state experienced when there is a discrepancy between the relationships one would like to have and that one perceives to have (Alberti, 2019). This condition does not so much concern the amount of time spent with other people as the quality of the relationships themselves.

In industrialized countries about one-third of people are affected by this condition, with one in severely affected, with these proportions constantly increasing (Cacioppo and Cacioppo, 2018). Loneliness is to such an extent a painful companion for many people that an editorial in the New York Times on the issue was entitled, “Is Loneliness a Health Epidemic?” (Klinenberg, 2018). Those who are most likely to report a significant feeling of loneliness tend to belong to the most vulnerable social groups, such as the young, the elderly, the poor, the chronically ill, and the mentally ill (Hawkins-Elder et al., 2018).

Importantly, loneliness has a profound impact on physical and psychological health, often leading to negative outcomes; loneliness and social isolation would appear to be associated with a reduction in lifespan like that caused by smoking 15 cigarettes a day, with a 27% increased risk of premature mortality (Holt-Lunstad et al., 2010). On the other hand, establishing strong relationships would lead to a reduced risk of mortality (Holt-Lunstad et al., 2017).

Several studies on the effects of loneliness on the health of the general population have been conducted over time. Loneliness is known to affect mental influence mental health, by leading to depression (Alpass and Neville, 2003; Cacioppo et al., 2006a; Hawkley and Cacioppo, 2010). Indeed, loneliness precedes mood disorder in time, proving to be a key factor in the onset of the disorder (Cacioppo et al., 2010): loneliness seems to mediate the anxiety-depression relationship, with loneliness potentially resulting from anxiety and subsequently being able to sequentially activate depressive symptoms (Ebesutani et al., 2015). The process behind this phenomenon is quite complex. It is believed that oxytocin and arginine vasopressin act as key mediators of social behavior in non-human mammals and human (Heinrichs and Domes, 2008). Oxytocin reduces behavioral and neuroendocrine responses to social stress and, as a result, may allow animals to overcome their natural aversion to close proximity and inhibit defensive behavior, thus facilitating approaches (Heinrichs and Domes, 2008). Seven primary emotional processes have been described by affective neuroscience: SEEKING, RAGE, FEAR, sexual LUST, maternal CARE, separation-distress PANIC/GRIEF and joyful PLAY (Panksepp, 1998; Zellner et al., 2011). Social loss, perhaps the biggest epidemiological determinant of depression, may promote deflection of mood through overactivity of separation-distress PANIC/GRIEF and hypoactivity of SEEKING networks (Panksepp, 1998, 2003; Zellner et al., 2011). Endogenous opioids, which may mediate attachment and separation distress *via* oxytocin pathways, contribute to initiating depressive cascades through decreased SEEKING (Gunnar and Quevedo, 2007; Heinrichs and Domes, 2008; Nolte et al., 2011). Thus, altered affective networks occurring in depression may explain psychological pain and dysphoria. Human health, including the need for social relationships, is largely driven by the endogenous opioid hormonal system (Johnson et al., 2014). Illness may result from disrupting this system.

Indeed, from a biomedical point of view, loneliness has been associated also with poor self-rated health (Stickley et al., 2013). Consistent with this, persistent loneliness has been associated with physical health problems (Newall et al., 2014) and sleep disorders (Cacioppo and Cacioppo, 2014). Moreover, it has also been linked with negative health habits, such as alcohol consumption (Stickley et al., 2013; Arpin et al., 2015) and smoking (Stickley et al., 2013).

In the neuropsychiatric field, loneliness has also been linked to obsessive-compulsive disorder (Timpano et al., 2014), social anxiety (Lim et al., 2016), and paranoia (Jaya et al., 2017). Loneliness has been associated with psychological distress (Stickley et al., 2013) but the most dramatic outcome of loneliness is suicide especially in populations at risk such as adolescents and old adults (Stravynski and Boyer, 2001; Morese et al., 2019a; Morese and Longobardi, 2020; Morese et al., 2020), an act almost constantly associated with the idea of being left alone and no longer able to receive help from anyone (De Leo and Diekstra, 1990). Indeed, strong associations among suicide ideation, parasuicide, and different ways of being lonely and alone were verified (Stravynski and Boyer, 2001). Importantly, the prevalence of suicide ideation and parasuicide increased with the degree of loneliness with differences between men and women (Stravynski and Boyer, 2001).

From a neuropsychological perspective, perceived social isolation (i.e., loneliness) is a risk factor for - and may contribute to - poorer overall cognitive performance, faster cognitive decline, poorer executive functioning, greater sensitivity to social threats, which is a confirmation bias in social cognition (Cacioppo and Hawkley, 2009). Therefore, social worlds tend to be perceived as threatening and punishing by lonely people (Cacioppo and Hawkley, 2009). Researchers have found that manipulating feelings of loneliness causes people to feel more anxious, fear negative evaluation, and act more coldly toward others (Cacioppo et al., 2006b), while also making them feel colder (Zhong and Leonardelli, 2008). Also, lonely people are more likely to form negative social impressions of others, and their expectations, attributional reasoning, and behavior toward others are less charitable than those of non-lonely individuals (Cacioppo and Hawkley, 2005). As a result of negative social expectations being validated by others, these expectations are reinforced and an individual is more likely to behave in ways that distance them from the very people they want to be close to better meet their social needs (Cacioppo and Hawkley, 2009). Hence, lonely individuals may perceive themselves as passive victims in their social world, yet they are active agents through their self-destructive interactions with others (Cacioppo and Hawkley, 2005).

Pandemic conditions have exacerbated these effects today as social distancing, fiduciary isolation, and quarantine have been imposed. The consequences on the elderly population are of particular interest (Morese et al., 2019b; Palermo, 2020a, 2021a; Amanzio et al., 2021).

## Loneliness and social cognition in old age

Since the second half of the 20th century, advances in healthcare and nutrition have led to an increase in the number of older adults in Western societies. Research in developmental and health care is challenged by this rapid increase. Aging involves new definitions of both self and relational issues in many developmental models (Blatt, 2008), since it concurs to psycho-physical, cognitive, and social impairment (Maylor et al., 2002). Moreover, as older adults age, they become increasingly confronted with the loss of loved ones.

Loneliness and social isolation are growing public health concerns in our aging society (Akoya et al., 2020). The prevalence of loneliness among the population is high. Eighty per cent of those under 18 years of age and 40% of those over 65 years of age report feeling lonely at least occasionally (Giné-Garriga et al., 2021). A growing number of older people in the EU are living alone: they form a particularly vulnerable group in society, with an increased risk of social exclusion or poverty (Eurostat, 2020). Seniors who live alone are often facing complex dynamics that rarely have an easy explanation: loneliness can occur due to the natural events of life, age-related challenges, and changes in social life (Savikko et al., 2005). The most common causes include family crisis, physical and motor limitations, death of many peers, widowhood, limiting housing conditions, increased use of communication through electronic devices rather than face-to-face.

Women suffer from this condition more often than men. Indeed, women tend to live longer than men and are more likely to experience any of the above-listed situations. In 2018, the share of older women (aged 65 years or more) in the EU-27 living alone was 40 % (Eurostat, 2020). Older women living alone reported severe difficulties and are hence more likely to be frail. Loneliness, together with age, chronic pathologies, and non-self-sufficiency, must be considered a risk factor for the frailty process. A longitudinal study on a sample of 1,600 respondents, found that 43% of the elderly lived in a condition of loneliness and, 6 years after the first interview, the researchers discovered that those who were lonely had a 45% higher risk of mortality, with a worsening of the quality of life and personal autonomy (Perissinotto et al., 2012).

According to large-scale surveys, loneliness increases the risk of mortality because it increases the presence of diseases as well as the use of pharmaceuticals and health services, thereby increasing the costs of public health (Holwerda et al., 2012; Banks et al., 2016).

Just for an example, the 4-years long-term effects of loneliness on health include increased blood pressure, depression, weight gain, smoking alcohol/drug use, alone time and decreased physical activity, cognition, heart health, sleep, stroke, and coronary heart disease (Berg-Weger and Morley, 2020).

**Figure 1:**
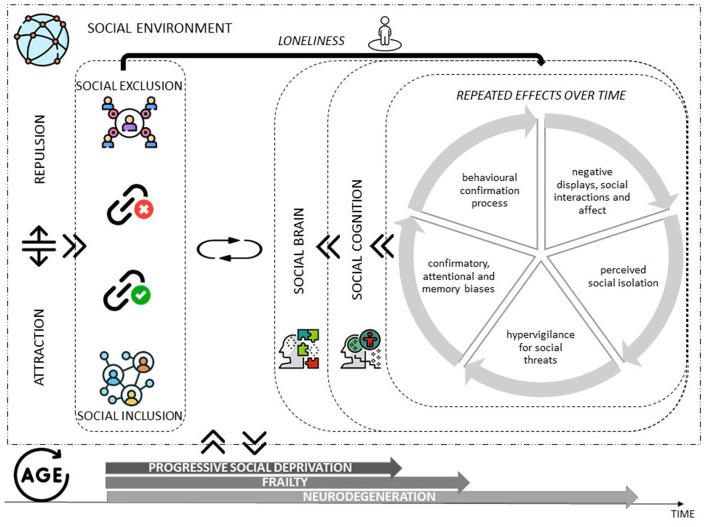
Theoretical model summarizing the relationships that exist between the dimensions of interest, the interactions between the individual and the social environment, and the dynamics of mutual interchange that affect the social cognition and social brain along the passage of time and the increasing age of the individual.

Loneliness also appears to be associated with cognitive decline and the onset of major neurocognitive disorder (Donovan et al., 2016; Rafnsson et al., 2017; Luchetti et al., 2020). It is precisely in neurocognitive diseases that the importance of social relations has been studied and how they can modulate and enhance the neural correlates of the circuits that create wellbeing in feeling that one belongs to a specific social group (Morese et al., 2018; Morese and Palermo, 2020) (Figure 1). Several studies have shown that the social brain network, associated with a positive feeling of wellbeing and pleasure (“warm glow”), is the one that is activated when people feel part of their communities and experience social support (Morese et al., 2016, 2019a; Lo Gerfo et al., 2019; Auriemma et al., 2020; Longobardi et al., 2020; Morese and Longobardi, 2022). This might suggest that older adults' ToM is driven by the retrieval of information relevant to isolation (Beadle et al., 2012).

Given the above, loneliness is a factor that should not be taken lightly in the daily lives of older people, since this existential condition may have affected their health by initiating an iatrogenic cascade process on all the body's physiological systems (Morese et al., 2019b; Palermo, 2021b). Elderly people living alone are undoubtedly made more frailty by a possible deprivation of a social support network (family or friends) that they can rely on in times of need. In many western countries, this issue is becoming increasingly important: this is undoubtedly due to demographic concerns linked to an aging population and increased life expectancy (Palermo, 2020a).

## An example of social brain disruption in aging: The case of Alzheimer's disease

As cognitive aging progresses, it is normal that there will be a progressive decline of social cognition (Moran et al., 2012). Performance during social-cognitive tasks was impaired and selectively accompanied by age-related decreases in the functional response of the dorsomedial prefrontal cortex. Based on these findings, age-related deficits in mentalizing are localized to circumscribed subregions of the default mode network, independent of tasks (Moran et al., 2012). The overlap between the social brain and the DMN have potential implications on neurodegenerative process. Indeed, several modifications of this network have been reported in in healthy older adults as well as in populations at risk for Alzheimer's disease (AD) (Mevel et al., 2011). In particular, decreased signal complexity in DMN nodes might contribute to cognitive decline in AD, which leads to even more dysfunctional DMN connectivity (Grieder et al., 2018) and potential effects on social cognition (Mars et al., 2012).

Concerning the perception of affiliation and isolation (i.e., loneliness), age-related similarities in the recruitment of brain regions involved in the ToM and self-referentiality (e.g., temporal pole, medial prefrontal cortex) are known (Beadle et al., 2012). Specifically, in response to isolation vs. affiliation imagery, older adults show greater recruitment than younger adults of the temporal pole, a region that is important for retrieving personally relevant memories used to understand the mental states of others (Beadle et al., 2012). A notable example of the effects of damage in that brain region is AD, which affects social functioning primarily through atrophy of the medial temporal lobe. Few studies have investigated ToM in AD patients using questionnaires, PET, and MRI/rs-fMRI. Yet no study has examined the neural correlates recruited during a social situation that involved the Theory of Mind. To measure the two components of the ToM, the attribution of emotions and intentions, Dodich et al. (2016) administered the Story-based Empathy task to patients with different neurodegenerative diseases. Both performances on the attribution of emotions and intentions were low in AD patients. Their poor performance may be due to a general cognitive decline, according to the authors (Dodich et al., 2016). The impairment of social skills in AD has a strong impact on interpersonal relationships, but to date, no neurophysiology studies deeply investigated the cognitive and emotional processes. Patients who were poorly involved in social activities and interactions tended to show more psychological and behavioral symptoms at baseline than socially involved patients (Arai et al., 2021). In addition, poor communication with family at baseline was associated with increased severity of psychiatric and behavioral disorders after 1 year (Arai et al., 2021). It, therefore, seems crucial to maximizing patients' involvement, as well as their opportunities for socialization and interaction, to prevent the exacerbation of symptoms over time (Arai et al., 2021).

An impairment of cognitive function may have an impact on loneliness for older people as it can hinder social interaction with family and friends, or interfere with judgements regarding relationship satisfaction (Burholt et al., 2017). The iatrogenic process tends to be self-sustaining. While alterations in social cognition occur with physiological aging, at the same time social isolation is itself inherently depressogenic and results in cascading effects on neurophysiological systems (Heinrichs and Domes, 2008; Nolte et al., 2011). Depressogenic mechanisms that emerge from loss of sociality include alterations in the stress axis, disinhibition of pro-inflammatory signals (Slavich and Irwin, 2014), particularly of the innate branch of the immune system (Cañas-González et al., 2020; Palermo, 2020a). The process contributes to the dysregulation of the stress axis, the decline in neurotrophins (also associated with increased inflammation), in opioids and oxytocin, which imay recursively contribute to a new reduction in neurotrophins (Heinrichs and Domes, 2008; Nolte et al., 2011).

The above fits into the context of the *social safety theory* (Slavich, 2020), which hypothesizes that the development and maintenance of friendly social ties is a fundamental organizing principle of human behavior and that threats to social security are a critical feature of psychological stressors that increase the risk of illness. It is likely that anticipatory neural-immune reactivity to social threat is highly conserved due to situations of social conflict, isolation, devaluation, rejection, and exclusion historically increasing risk of physical injury and infection (Slavich, 2020). Survival ultimately depends on humans' ability to elaborate symbolically and respond to a potential danger situation, which is a neurocognitive and immunological function (Slavich, 2020). Positive and negative social experiences can be explained by the social safety theory on a biological and evolutionary basis, allowing us to explain why certain stressors are particularly harmful. The framework also provides a multilevel approach to explore the biopsychosocial determinants of health and aging disparities, physical and cognitive frailty, and interpersonal behavior impairment (Slavich, 2022).

Social isolation – through the biological routes described by the *social safety theory*- may contribute, through the primary induction of depression, to the increased risk of AD (Kuo et al., 2020; Drinkwater et al., 2021). Indeed, socially isolated people had lower volume in the brain's gray matter in brain regions involved with learning and thinking. Social isolation counts for a 26 percent increase in the likelihood of dementia onset (Shen et al., 2022). Due to their complex relationship, the association between dementia and late-life depression is still unclear (Kuo et al., 2020). Indeed, it has been proposed that to understand the pathogenic mechanisms of AD, it is necessary to consider its multifactorial nature considering all together risk factors such as hypertension, social engagement, obesity, education level, or physical inactivity (Kuo et al., 2020). There could therefore be “softer” secondary pathways to neurodegeneration even for those who do not become formally depressed, but simply for those who were isolated and for dysphoric without being formally depressed. Just as an example, there is a well-known association between social isolation, loneliness, and cardiovascular disease (Golaszewski et al., 2022). Epidemiological studies report an independent association between dementia and cardiovascular disease, suggesting that stroke and dementia may share overlapping molecular mechanisms (Stakos et al., 2020; Leszek et al., 2021). Indeed, the pathogenesis of cardiovascular disease and AD is influenced by low-grade inflammation (Stakos et al., 2020). In particular, one of the major risk factors found to affect the cardiovascular system as well as the nervous system is ApoEε4 (Leszek et al., 2021).

## Conclusions

Attachment theory highlights the existence in humans of an innate need to seek protective closeness and intimacy with significant figures during times of crisis, suffering, need or distress. However, this innate need is supplemented from an early age with experiences resulting from the environment in which the individual is immersed. Accordingly, the human tendency to desire and seek the closeness of attachment figures corresponds to an innate schema whose full operationalization is dependent on concrete experiences in relationships. In this sense, children's first relationships with caregivers influence the development of internal working models, expectations of themselves and others, and provide the basis for learning and social interactions. Being able to experience meaningful affective relationships and the quality of these relationships is essential both for maintaining self-confidence and emotional stability in the elderly and for coping with the traumas associated with neuropsychological and physical frailty. How the elderly person becomes available for the type of care they will receive depends on the relationship they have with their reference figures from the past. People who have a history of insecure attachments may be less likely to trust caregivers and medical personnel.

Often the lack of meaningful interpersonal relationships in old age results in loneliness, which some studies show is related to particular attachment styles (Cicirelli, 2010). It is not simply “being alone” that is an indicative or predictive factor of loneliness. Instead, the perception of loneliness is influenced by expectations about oneself and others. These expectations are the results of internal working models, which function in accordance with the person attachment style.

Social cognition, in turn, is associated with social inclusion; impairment of social cognition associated with social brain dysfunctioning is associated with loneliness (Figure 1). One might ask whether experiences of loneliness have increased and whether loneliness is a characteristic of modern societies, or it has always existed to a certain extent. It is not possible to answer this question because there is still no international standard for the definition of loneliness (and therefore the available data often do not distinguish between social isolation, living alone, and loneliness), and because past epidemiological investigations did not pay attention to this issue. However, the general feeling is that the phenomenon is increasing and has important consequences for the psychophysical health of individuals, especially the elderly. A modifiable factor, loneliness, can be ameliorated before the development of severe impairment or neurodegenerative disorders likely-due-to-AD. Indeed, inflammation-related diseases and viral infections are among the most prevalent forms of morbidity and mortality associated with this multilevel biological threat response due to social stress and according to the *social safety theory* (Slavich, 2022). Therefore, this theoretical paper's primary aim is to underline the importance of the social neuroscience perspective to study the social brain and social cognition in the older population, to draw useful indications for preventing the iatrogenic effects of isolation on psychophysical wellbeing and acceleration of neurodegeneration-related processes.

## Author contributions

RM conceived the content of the article, participated in writing and supervised the manuscript for the parts falling within her competence. SP conceived the content of the article, participated in writing the first draft of the manuscript, wrote the last version, produced the infographics and supervised the entire manuscript.

## Funding

Open access funding was provided by the Università Della Svizzera Italiana.

## Conflict of interest

The authors declare that the research was conducted in the absence of any commercial or financial relationships that could be construed as a potential conflict of interest.

## Publisher's note

All claims expressed in this article are solely those of the authors and do not necessarily represent those of their affiliated organizations, or those of the publisher, the editors and the reviewers. Any product that may be evaluated in this article, or claim that may be made by its manufacturer, is not guaranteed or endorsed by the publisher.
