# Erratum: Feelings of loneliness and isolation: Social brain and social cognition in the elderly and Alzheimer's disease

**DOI:** 10.3389/fnagi.2023.1180611

**Published:** 2023-03-15

**Authors:** Frontiers Production Office

**Affiliations:** Frontiers Media SA, Lausanne, Switzerland

**Keywords:** elderly, loneliness, social isolation, social brain, social cognition, social neuroscience, mental health, Alzheimer's disease

An omission to the funding section of the original article was made in error. The following sentence has been added: “Open access funding was provided by the Università Della Svizzera Italiana.”

The original article has been updated.

